# Dynamical behaviors, circuit design, and synchronization of a novel symmetric chaotic system with coexisting attractors

**DOI:** 10.1038/s41598-023-28509-z

**Published:** 2023-02-02

**Authors:** Haitao Qiu, Xuemei Xu, Zhaohui Jiang, Kehui Sun, Can Cao

**Affiliations:** 1 School of Physics and Electronics, Central South University, Changsha, 410083 China; 2 School of Automation, Central South University, Changsha, 410083 China

**Keywords:** Nonlinear phenomena, Statistical physics, thermodynamics and nonlinear dynamics

## Abstract

In this paper, we introduce a novel three-dimension chaotic system with strange characteristic by applying construction of a 3D chaotic circuit method. Multiple equilibria and abundant coexisting attractors exist in this system. A mathematical model is developed and detailed stability analyses for equilibrium points are executed with obtaining significant results of the period-doubling bifurcation patterns confirmed by phase plane plots and Lyapunov exponent spectra. By varying the initial value and unique controlled parameter, the double-scroll chaotic attractor is broken up into a pair of symmetric singular attractors. Then, the local basins of attraction are investigated concerning the initial condition. Next, the circuit synthesis results generated by Multisim simulation tool validate the self-excitation characteristics of this system. Finally, the feedback control technique is used to study difference synchronization of this system. Main conclusions prove the validity and reliability of difference synchronization.

## Introduction

In 1963, “chaos” was first discovered in numerical experiments on weather dynamics^1^. It is a seemingly random movement, meaning that random-like behaviors occur without addition of any random factors, in deterministic nonlinear systems. As a branch of nonlinear science, chaos theory is widely applied in medical diagnosis^2^, economy^3^, image encryption^4–6^, neural network^7^, weak signal detection^8,9^, secure communication^10^, etc. Chaotic characteristics, depending greatly on initial conditions and system parameters, illuminate many interesting complicated nonlinear phenomena. Since the existence of coexistence attractors provides a variety of optional steady states for systems, it gradually become a research hotspot in recent years. Coexistence attractors indicate that two or more attractors are generated in different parameters and initial conditions^11^. A classic example is that the butterfly attractor of the Lorenz system is broken into a pair of symmetric singular attractors in a previously unexplored parameter space region^12^. Kengne et al. proposed a three-dimensional Jerk system with cubic nonlinear terms and found that the coexistence of attractors is closely related to parameter variations^13^. Bao et al. constructed a memristor chaotic circuit and observed the coexistence of an infinite number of attractors^14^. The chaos singularities and instabilities can be described by hidden attractors and self-excited attractors. A self-excited one means that the basin of attraction is excited from unstable equilibria^15^. The other is defined as an attractor with multiple equilibrium points and stable equilibrium states, or without any equilibrium^16,17^. Until now, nonlinear electronic circuits with complex dynamical behaviors, such as self-excited chaotic oscillations, hidden oscillations, and the behaviors of coexisting multiple attractors^18^ have been explored theoretically and numerically.

With booming Internet techniques, the security of information transmission is required of great significance to the public. Nowadays, chaos synchronization has been successfully applied in secure communication^19,20^. On the basis of proposing a chaotic self-synchronization method and realizing synchronization of two chaotic systems^21^, various chaos synchronization schemes have been developed, such as complete synchronization^22^, anti-synchronization^23^, generalized synchronization^24^, phase and anti-phase synchronization^25,26^, projective synchronization^27^, combination synchronization^28,29^, combination–combination synchronization^30^, and compound synchronization^31^. Firstly, reference^32^ introduced a difference synchronization method, which realizes synchronization between two driving systems and one response system by using the method of linear weighted combination. The flexible selection of scaling factor makes the geometric topology of coupled system more complex and the prediction of the path to chaos more difficult for better secure communication performance. In order to realize the above chaotic synchronization schemes, a large amount of control techniques have been developed, such as linear and nonlinear feedback control^33^, sliding mode control^34^, active control^35^, adaptive control^36^, and neural network^37^. Du et al. derived a criterion for finite-time synchronization of fractional order memristor-based neural networks with time delay^38^. Wang et al. proposed a memristive synapse control method for designing multi-structure chaotic attractors and investigated the synchronization issue of memristive neural networks via an aobserver-based controller^39,40^.

In this research, we intend to propose a three-dimensional nonlinear chaotic system with multiple stable states whose stability and equilibrium points can be easily controlled. Unlike only quadratic nonlinear terms in most existent systems, our system makes the dynamic characteristics more complex by adding a cubic nonlinear term. In addition, by introducing stable variable parameters, we solve the Jacobin matrix and plot the portraits of the characteristics of the stable state to obtain the details of the unstable focus, stable nodes, and stable points. Moreover, we employ a bifurcation diagram coinciding with a spectrum of the largest Lyapunov exponents to explore chaos behaviors and coexisting attractors. Specifically, the contribution of this paper is mainly four fold: (1) The designed system is adaptable to a self-excited oscillator in an integrated system; (2) circuit of chaotic system is designed and simulated by Multisim software, which can effectively verify the numerical simulation results; (3) linear feedback control is suitable for chaotic systems with cubic nonlinearity, used in difference synchronization; (4) based on synchronization schemes, our results are practical in secure communication.

## System description

### Mathematical model

In 2013, a series of three-dimensional chaotic systems with quadratic nonlinearities were proposed by Jafari and Sprott^41^, where the mathematical models of Sprott A system and NE8 system can be expressed by following autonomous differential Eqs. (1) and (2)

Sprott A system:

1 $$\begin{array}{c}\left\{\begin{array}{l}\dot{{x}_{1}}={y}_{1},\\ \dot{{y}_{1}} ={-x}_{1}-{y}_{1}{z}_{1}\\ \dot{{z}_{1}}={y}_{1}^{2}-a.\end{array},\right.\end{array}$$

NE8 system:

2 $$\begin{array}{c}\left\{\begin{array}{l}\dot{{x}_{2}}={y}_{2},\\ \dot{{y}_{2}}={-x}_{2}-{y}_{2}{z}_{2},\\ \dot{{z}_{2}}={0.5x}_{2}^{2}+{x}_{2}{y}_{2}-b,\end{array}\right.\end{array}$$

where ${x}_{1}$, ${y}_{1}$, ${z}_{1}$ and ${x}_{2}$, ${y}_{2}$, ${z}_{2}$ are state variables, $a\mathrm{ and }b$ are constant parameters.

**Figure 1:**
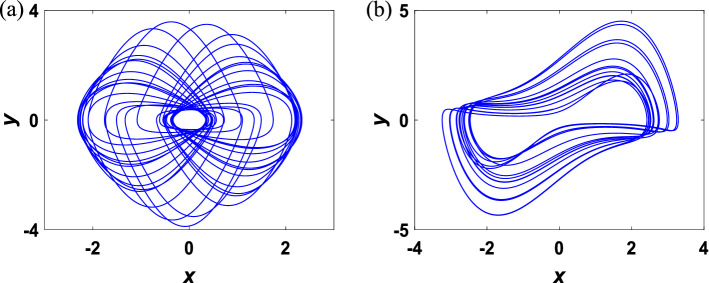
Chaotic attractors of systems (1) and (2) with (**a**) parameter *a* = 1 and initial condition (− 0.1, − 1, 0.3). (**b**) Parameter *b* = 1.47 and initial condition (0, 0.1, 0).

Remarkably, multiple stability exists in the above systems with no equilibrium, indicating the existence of coexisting attractors^42^. Intuitively, Fig. 1 shows chaotic attractors of the systems with parameters *a* = 1 and *b* = 1.47. Then, a new 3D chaotic system is constructed from Sprott A system by adding a cubic nonlinear term. Consequently, the corresponding mathematical model of this system is formulated as

3 $$\begin{array}{c}\left\{\begin{array}{l}\dot{x}=y-2xz,\\ \dot{y}=-x+0.5\left(1-{x}^{2}\right)y-0.5yz,\\ \dot{z}=0.1xy+\nu {x}^{2}-0.8,\end{array}\right.\end{array}$$

where $x$, $y$, $z$ are state variables, and $v$ is a constant parameter.

### Equilibrium points and stability analysis

When parameter $v$ denotes an adjustable variable, it is easy to deduce equilibrium points of system (3) by solving $\dot{x}=0$, $\dot{y}=0$, $\dot{z}=0$:

The equilibrium points can be expressed as $S=(\widehat{x},\widehat{y},\widehat{z})$, where

4 $$\begin{array}{c}\left\{\begin{array}{l}\widehat{y}=\frac{8}{\widehat{x}}-10v\widehat{x},\\ \widehat{z}=\frac{4}{{\widehat{x}}^{2}}-5v.\end{array}\right.\end{array}$$

From Eq. (4), it is trivial to make out that $\widehat{y}$ and $\widehat{z}$ are subject to state variable $\widehat{x}$ and parameter $v$. Thus, $S$ changes with parameter $v$. By linearizing (3) around the equilibrium point, the Jacobin matrix can be expressed as:

5 $$\begin{array}{l}J=\left[\begin{array}{ccc}-2\widehat{z}& 1& -2\widehat{x}\\ -1-\widehat{x}\widehat{y}& 0.5\left(1-{\widehat{x}}^{2}-\widehat{z}\right)& -0.5\widehat{y}\\ 0.1\widehat{y}+2v\widehat{x}& 0.1\widehat{x}& 0\end{array}\right].\end{array}$$

The characteristic equation can be derived as

6 $$\begin{array}{c}{\lambda }^{3}{+{a}_{1}\lambda }^{2}+{a}_{2}\lambda +{a}_{3}=0,\end{array}$$

where $\lambda$ is the eigenvalues of Eq. (6) and

7 $$\begin{array}{l}\begin{array}{c}{a}_{1}=2.5\widehat{z}-0.5\left(1-{\widehat{x}}^{2}\right),\\ {a}_{2}=4v{\widehat{x}}^{2}+1.25\widehat{x}\widehat{y}-\left(1-{\widehat{x}}^{2}\right)\widehat{z}+{\widehat{z}}^{2}+1,\\ {a}_{3}=2v{\widehat{x}}^{4}-0.1{\widehat{x}}^{3}y-\left(0.2+2v\right){\widehat{x}}^{2}+2v\widehat{z}{\widehat{x}}^{2}+0.2\widehat{x}\widehat{y}\widehat{z}+\left(v-0.1\right)\widehat{x}\widehat{y}+0.05{\widehat{y}}^{2}.\end{array}\end{array}$$

**Figure 2:**
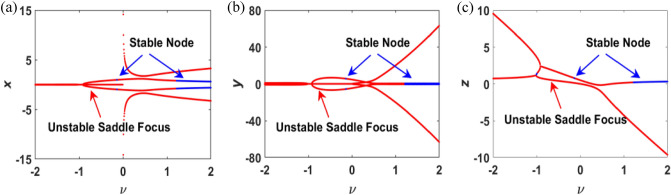
Numerically simulated equilibrium points and stability analysis with $\nu \in [-\,\mathrm{2,2}]$.

The constant parameter $v$ changes in the range of [$-$ *c*, *c*] with the time evolution, thus we can get the values of $\widehat{x}$, further $\widehat{y}$, and $\widehat{z}$. To explore the exact points and stability of the equilibrium point, we set the boundary of parameter A as [− 2, 2], then the results are depicted in Fig. 2 intuitively.

In order to avoid solving numerical solution of eigenvalues of the Jacobi matrix, stability of the equilibrium point is described by phase diagram trajectory, due to existence of quadratic and cubic nonlinear terms. According to the Routh–Hurwitz criterion, the stability of equilibrium points can be estimated by solving Eq. (6). In this chaotic system, equilibrium points are classified into two types: unstable saddle focus and stable node. Negative real eigenvalues or complex eigenvalues with negative real parts are stable nodes. On the contrary, those positive real eigenvalues or complex eigenvalues with positive real parts are unstable nodes. Those eigenvalues are saddle nodes if the roots are real eigenvalues with different signs.

From Fig. 2, it can be observed that the locus of equilibrium points changes with parameter $v$ in range of $[-\,2, 2]$ as time goes on. The red line denotes the unstable saddle focus and the blue line denotes the stable nodes. The three diagrams labeled (a), (b), and (c) in Fig. 2 represent values of three dimensions of the equilibrium points.

### Dynamical analysis

#### Route to chaos

**Figure 3:**
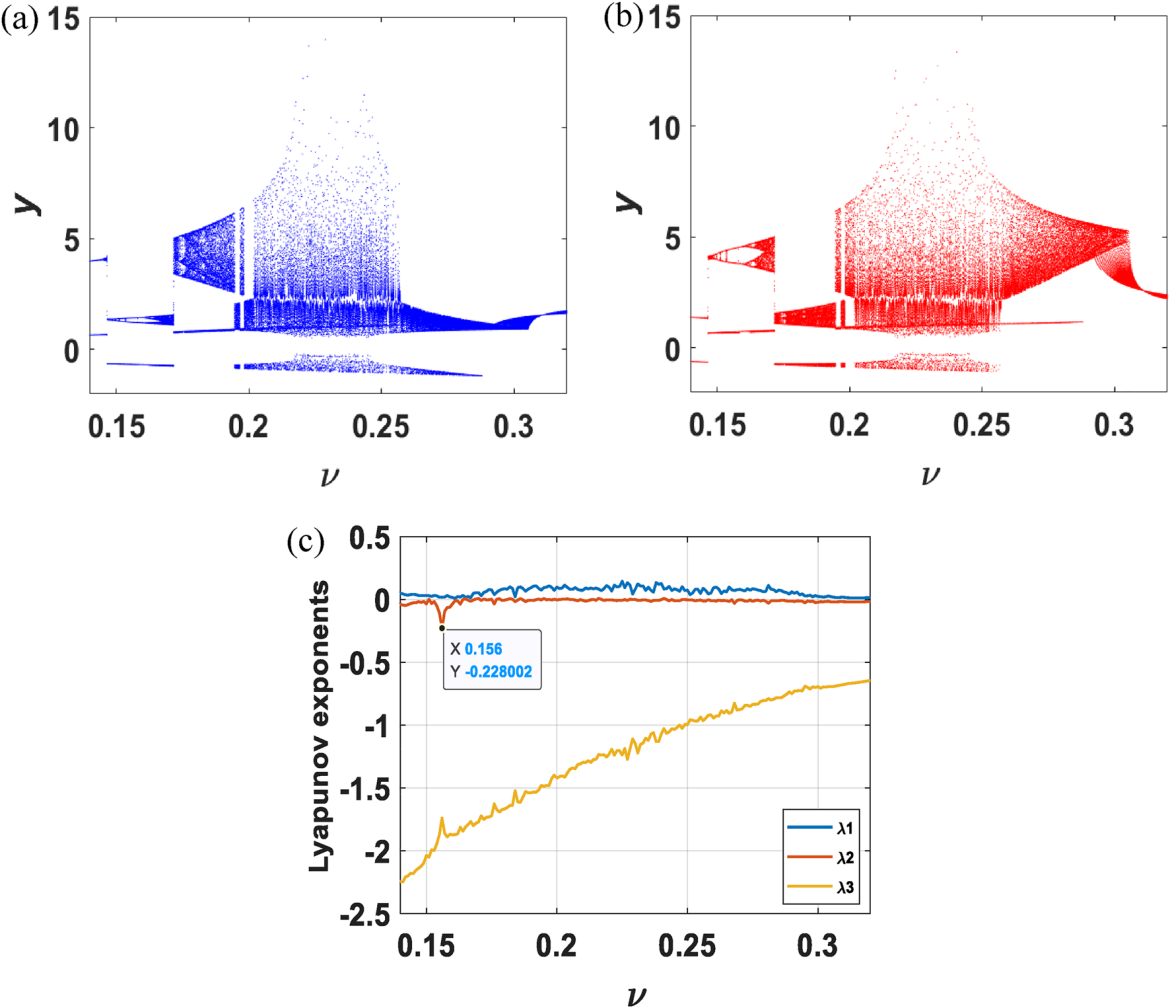
For the initial values (0.1, 2, 0.1) and (− 0.1, − 2, 0.1), the bifurcation diagram and Lyapunov spectrum of system (3) as $v$ varies.

**Figure 4:**
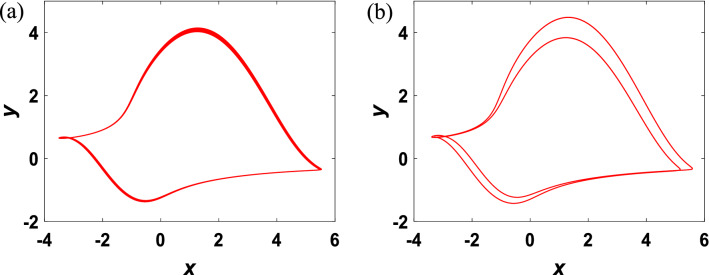
For the initial values (− 0.1, − 2, 0.1), the phase diagrams of system (3) in *x*–*y* plane: (**a**) quasi-periodic state with ${\nu }_{1}=0.147$. (**b**) Period state with ${\nu }_{1}=0.156$.

By changing initial conditions and tuning parameters, phase trajectories and dynamics behaviors are investigated qualitatively. Bifurcation diagrams versus $v\in [0.14, 0.32]$ from initial conditions ${x}_{01}=\left(0.1, 2, 0.1\right), {x}_{02}=(-\,0.1,-\,2, 0.1)$ are depicted in Fig. 3a,b, with proving the existence of chaotic attractors of various trajectories, limit cycles of different periods, period-doubling bifurcation, and coexistence bifurcation in the system. Different from the systems in most papers, the system developed in this paper is a period-doubling bifurcation of periodic and quasi-periodic states. According to Fig. 3b, as setting the parameters ${\nu }_{1}=0.147$ and ${\nu }_{2}=0.156,$ respectively, the corresponding attractors are shown in Fig. 4, where the Lyapunov exponents calculated by the algorithm (A. Wolf, J. B. Swift) are ${\lambda }_{11}=0.0153,{\lambda }_{12}=- \,0.0159,{\lambda }_{13}=-\, 2.1108$ and ${\lambda }_{21}=0.0040,{\lambda }_{22}=-\, 0.2545,{\lambda }_{23}=-\, 1.7009$, indicating that the system is in quasi-periodic and periodic states, respectively. In various conditions, the system undergoes Hopf bifurcation and enters a continuous oscillation state, and then falls into chaos through period-doubling bifurcation. A normal oscillating behavior suddenly appears or disappears, leading to emergence of coexisting attractors, reflecting the complexity of nonlinear characteristics of the system.

The largest Lyapunov exponent is an important quantitative index to measure dynamic characteristics. It represents the average exponential rate of the convergence or divergence of a system between adjacent orbits in phase space. A critical threshold of the system state can be obtained indirectly from a joint state of the largest Lyapunov exponents of the system. When $v$ is varied from 0.14 to 0.32, the single-parameter Lyapunov exponent spectrum is drawn in Fig. 3c. We can see that the marker point indicates the periodic state of the system resulting from the sign of three Lyapunov exponents is $(0,-,-)$. It is worth noting that the bifurcation diagram coincides with the spectrum of the largest Lyapunov exponents. In particular, the algorithm employed in this work for determining the largest Lyapunov exponents was proposed in (A. Wolf, J. B. Swift).

#### Coexisting attractors

**Figure 5:**
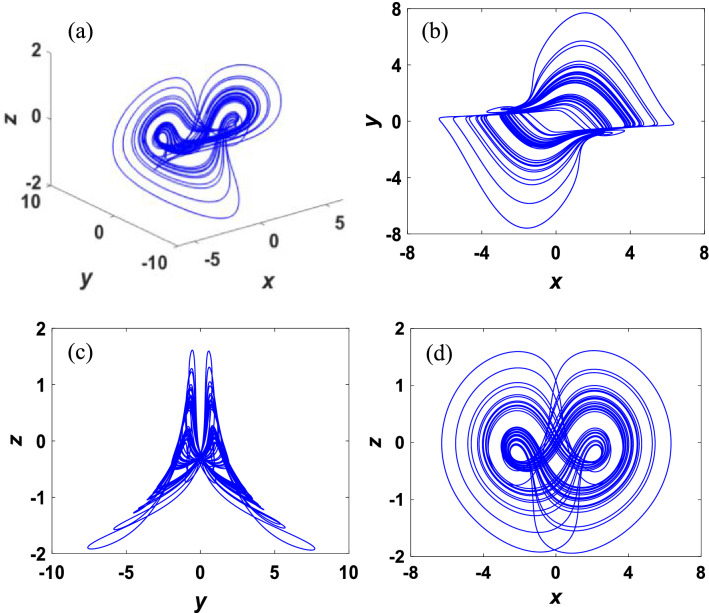
Chaotic attractors of the system (3) with parameter $\nu =0.21$ and initial condition (0.1, 2, 0.1).

Coexisting attractors provide multiple optional steady states for the system to respond to different requirements. For parameter $v=0.21$ and initial condition (0.1, 2, 0.1), the double-scroll chaotic attractor is depicted in Fig. 5. The Lyapunov exponents of the system are ${\lambda }_{1}=0.0864,{\lambda }_{2}=-0.0037,{\lambda }_{3}=-\,1.3122$. It can be derived that the summation of LEs is negative:

8 $$\begin{array}{c}{\lambda }_{1}+{\lambda }_{2}+{\lambda }_{3}=-\,1.2295<0,\end{array}$$

which shows dissipation of system. The corresponding Lyapunov exponent dimension is

9 $$\begin{array}{c}{D}_{\lambda }=j+\frac{{\sum }_{i=1}^{j}{\lambda }_{i}}{\left|{j}_{j+1}\right|}=2+\frac{{\lambda }_{1}+{\lambda }_{2}}{\left|{\lambda }_{3}\right|}=2.063,\end{array}$$

where variable $j$ satisfies ${\sum }_{i=1}^{j}{\lambda }_{i}>0$ and ${\sum }_{i=1}^{j+1}{\lambda }_{i}<0$. The symmetric strange attractor can be observed because the Lyapunov exponent dimension is fractional and system dissipation.

**Figure 6:**
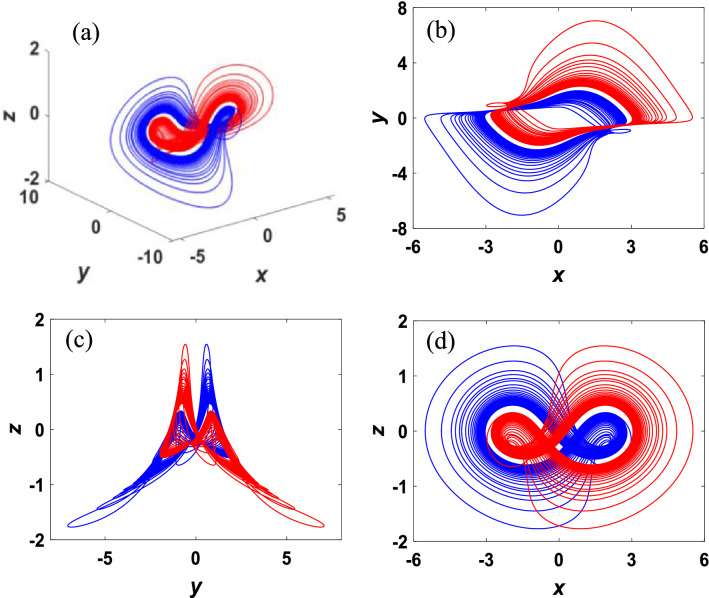
A pair of symmetry singular attractors of the system (3) with parameter $\nu =0.26$ and initial condition ($\pm$ 0.1, $\pm$ 2, 0.1).

Change the parameter $v=0.26$, then two independent attractors are yielded in the system (3) with initial values (± 0.1, ± 2, 0.1), as shown in Fig. 6. The red line denotes the attractor with initial condition ${x}_{01}=\left(0.1, 2, 0.1\right)$ and the blue line denotes the attractor with initial condition ${x}_{02}=(-\,0.1, -\,2, 0.1)$. It can be verified that the attractors are chaotic as they have the same positive maximum Lyapunov exponent ${\lambda }_{1}=0.0758$ and fractal Lyapunov dimension ${D}_{\lambda }=2.078$. Accordingly, the double-scroll chaotic attractor in Fig. 5 is broken into two singular attractors. It is easy to verify that the two strange attractors have rotational symmetry about the z-axis.

**Figure 7:**
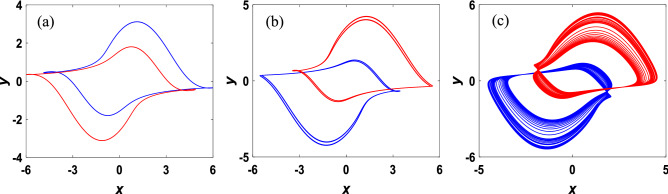
The phase portraits of coexisting symmetric attractors in the x–y plane with initial conditions (± 0.1, ± 2, 0.1): (**a**) $\nu =0.1$. (**b**) $\nu =0.15$. (**c**) $\nu =0.3$.

The period-doubling bifurcation and coexistence bifurcation can be visually illustrated by generating the phase portraits of the system (3) with initial conditions (± 0.1, ± 2, 0.1). As shown in Fig. 7, system (3) performs period-1, period-2, and chaos respectively for $v=0.1, 0.15, 0.3$*,* implying that the process of chaos produced by period-doubling bifurcation is accompanied by coexistence bifurcation.

**Figure 8:**
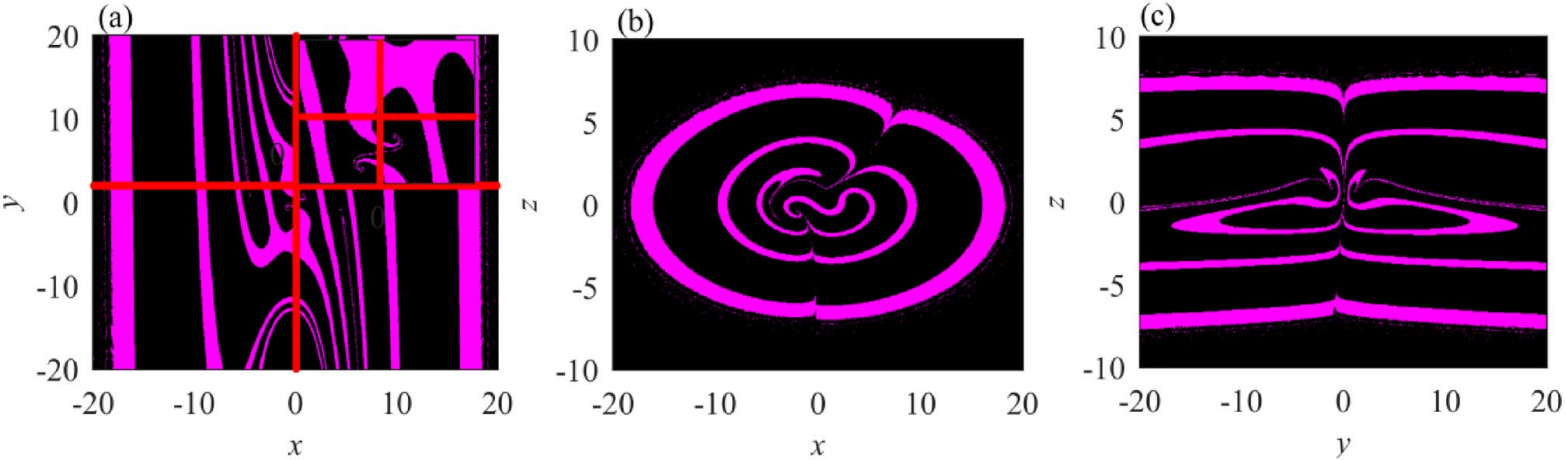
The local basins of attraction in three different planes. (**a**) The $x\left(0\right)-y\left(0\right)$ plane with $z\left(0\right)=0.1$. (**b**) The $x\left(0\right)-z\left(0\right)$ plane with $y\left(0\right)=2$. (**c**) The $y\left(0\right)-z\left(0\right)$ plane with $x\left(0\right)=0.1$.

For the coexisting symmetric attractors illustrated in Fig. 7c, the corresponding attractor basins three different planes are shown in Fig. 8, where the purple region corresponds to a pair of symmetric strange attractors, and the black region represents the initial condition for generating unbounded orbits. The basin has expected z-axis rotation symmetry and a complex fractal structure.

The initial condition can be regarded as an invariant measure for the classification of dynamic behavior. For chaotic systems, slight differences among initial conditions can cause large differences in solutions over time. If the bounded behaviors are found, the dynamic behaviors of chaotic spiking, stable resting, and periodic spiking are afterward classified by measuring the attractor sizes. According to the local basins of attraction, the stability of the initial condition-dependent dynamic behaviors can be distinguished evidently. The initial conditions are considered as $(x\left(0\right), y\left(0\right), 0.1)$, $(x\left(0\right), 2, z(0))$ and $(0.1, y\left(0\right), z(0))$ while the parameter is kept as $\nu =0.3$. Figure 8 portrays the attraction basin in $x\left(0\right)-\mathrm{y}(0)$, $x\left(0\right)-z(0)$, and $y\left(0\right)-z(0)$, respectively. Figure 8a demonstrates that two red lines are parallel to the x-axis and y-axis. And the intersection of two straight lines which indicates the initial state $(0.1, 2, 0.1)$ is located in the black regions meanwhile the parameter of system $v=0.3$. The initial condition $(0.1, 2, 0.1)$ demonstrates that initial-dependent behavior of system performed as unstable chaos. Therefore, it can be deduced from this phenomenon that long-term dynamical behaviors are associated with initial conditions. And it leads to emergence of bi-stability as well as consisting of unstable chaos and stable points. According to Fig. 8b, the local basin of attraction changes in a circular orbit, which is not continuous but discrete, indicating that the chaotic oscillator transitions from one oscillating state to another state. This is similar to the energy level transition in physics. Such basin of attraction is rare in the proposed chaotic systems. The basin of attraction in Fig. 8c changes in a discrete strip orbit, showing more abundant oscillation characteristics, so it can enhance the security of synchronous communication. Notably, the attractors of the proposed system are self-excited rather than hidden because their basins of attraction include multiple unstable equilibrium fields.

**Figure 9:**
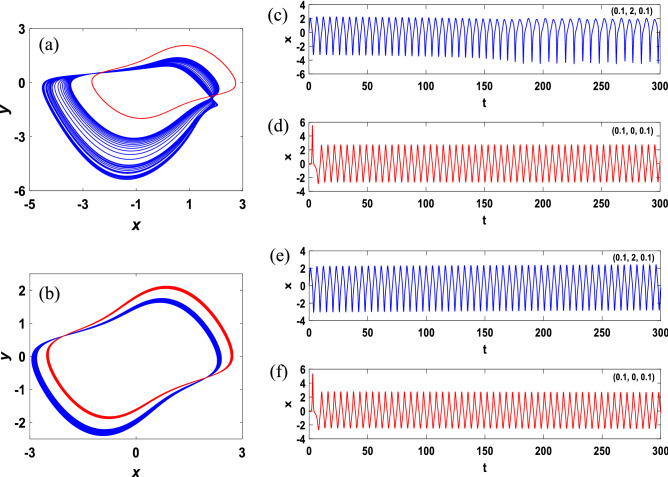
Two types of coexisting asymmetric attractors and time series of the variable $x$ emerged from the initial values (0.1, 2, 0.1) and (0.1, 0, 0.1).

In addition to the coexistence of symmetric attractors, when $v=0.3, 0.32$ with the initial values (0.1, 2, 0.1) and (0.1, 0, 0.1), two types of asymmetric coexistence attractors phase diagrams and the corresponding time series of variable $x$ are shown in Fig. 9. The coexistence of chaotic attractors and limit cycles and quasi-periodic coexistence can also be observed in Fig. 9a,b respectively. The time series of the variable $x$ corresponding to Fig. 9a are illustrated in Fig. 9c,d. Similarly, the relationship of Fig. 9b,e,f is the same as the former. From Fig. 9d,f, a transient effect is produced when the oscillation is started, and stability is achieved after some time.

### Circuit design

To investigate dynamics and confirm feasibility of a theoretical chaotic model, the circuit implementation of their corresponding mathematical models^43,44^ is commonly used. It is practical to use electronic circuits emulating chaotic systems because of their extensive application in engineering. Hence, the electronic circuit of the new chaotic system (3) is designed and verified in this section.

Numerous studies^45^ have pointed out that the fractional-order operators cannot be realized directly under the standard definition of fractional-order differ integral in time-domain simulations. If circuit is designed directly according to system equations, the circuit will not work normally. By applying operational-amplifier approach^46^, the variables’ state of the system (3) should be scaled down to realize strange attractors. According to system Eq. (3), scaling variables $X$, $Y$, $Z$ are settled as $X=x/2$, $Y=y/2$, $Z=z/4$, respectively. Where $x$, $y$, and $z$ are the state variables in system Eq. (3). The system can be implemented by utilizing common electronic components which are resistors, capacitors, analog multipliers, and operational amplifiers.

**Figure 10:**
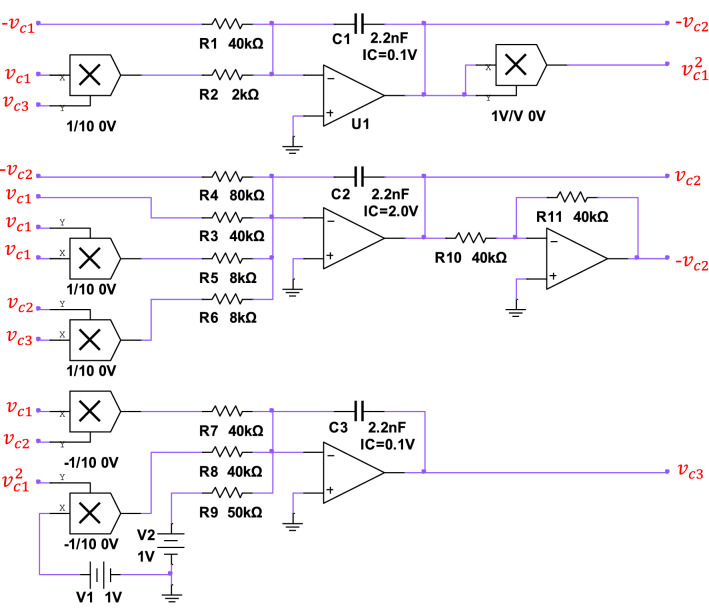
Implementation of circuit chaotic system.

By applying Kirchhoff laws to the electronic circuit, the corresponding circuit state equation set of the proposed novel chaotic system can be expressed as

10 $$\begin{array}{c}\left\{\begin{array}{l}\frac{d{\nu }_{C1}}{dt}=\frac{1}{{R}_{1}{C}_{1}}{v}_{C2}-\frac{1}{10{R}_{2}{C}_{1}}{v}_{c1}{v}_{c3} ,\\ \frac{d{v}_{c2}}{dt}=-\frac{1}{{R}_{3}{C}_{2}}{v}_{c1}+\frac{1}{{R}_{4}{C}_{2}}{v}_{c2}-\frac{1}{10{R}_{5}{C}_{2}}{v}_{c1}^{2}{v}_{c2}-\frac{1}{10{R}_{6}{C}_{2}}{v}_{c2}{v}_{c3}\\ \frac{d{v}_{c3}}{dt}=-\frac{1}{10{R}_{7}{C}_{3}}{v}_{c1}{v}_{c2}+\frac{1}{10{R}_{8}{C}_{3}}{v}_{C1}^{2}-\frac{1}{{R}_{9}{C}_{3}}{V}_{\alpha } ,\end{array} \right.,\end{array}$$

where ${v}_{c1}$, ${v}_{c2}$, and ${v}_{c3}$ are the voltages across the capacitors ${C}_{1}$, ${C}_{2}$, ${C}_{3}$, respectively. And ${V}_{\alpha }$ is a stable DC voltage source to implement the constant in a numerical system (3). Noticeably, the only parameter $v$ in (3) can be set by manually tuning resistor ${R}_{8}$. It can be inferred that three scaling variables $X$, $Y$, and $Z$ represent the voltage across the corresponding capacitors, respectively. The complete circuit is implemented on the electronic simulation platform Multisim, where Fig. 10 describes the designed circuit implemented by Multisim simulation. To realize a nonlinear chaotic system, the whole circuit contains three capacitors, eleven resistors, six multipliers, and four operational amplifiers. It can be noticed that three multipliers are configured as 1/10, the other two are configured as -1/10, and the last one is 1/1. The values of all electronic components in Fig. 10 are determined as follows: ${R}_{1}={R}_{3}={R}_{7}=$ 40 $\mathrm{k\Omega }$, ${R}_{2}=$ 2 $\mathrm{k\Omega }$, ${R}_{4}=$ 80 $\mathrm{k\Omega }$, ${R}_{5}={R}_{6}=$ 8 $\mathrm{k\Omega }$,${R}_{8}=$ 13.33 $\mathrm{k\Omega }$, ${R}_{9}=$ 50 $\mathrm{k\Omega }$, ${C}_{1}={C}_{2}={C}_{3}=$ 2.2 $\mathrm{nF}$, and ${V}_{\alpha }=$ 1 V, where ${R}_{8}$ is a variable resistor, and its resistance value needs to be adjusted to achieve different states. Other resistance and capacitance parameters in the chaotic circuit are not unique. The circuit depicted in Fig. 10 is just one implementation of the oscillator, which depends on different application scenarios. For example, in the PCB layout, it is necessary to adjust the appropriate capacitance position and parameters to reduce the influence of parasitic capacitance on the overall circuit.

**Figure 11:**
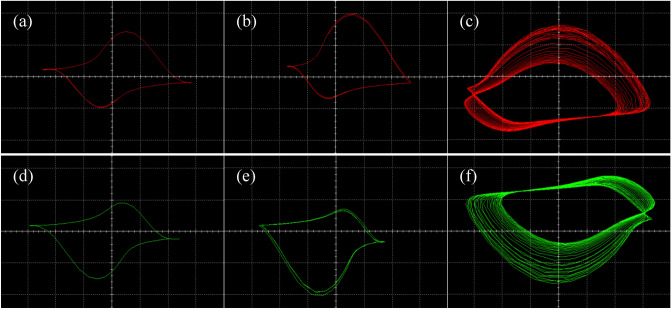
The symmetric coexisting attractors obtained from the designed circuit with the channels of ${v}_{c1}$ and ${v}_{c2}$.

The simulation results, which are phase portraits in the *x–y* plane of the system, are shown in Fig. 11 with connecting the channels of ${v}_{c1}$ and ${v}_{c2}$ in the circuit to oscilloscope. When the resistance is adjusted to ${R}_{8}=$ 40 $\mathrm{k\Omega }$, the phase portraits of limit cycle are illustrated in Fig. 11a,d with the corresponding parameter *v* = 0.1. Figure 11b,e depict the attractors of period-2 by setting the resistance ${R}_{8}=$ 26.67 $\mathrm{k\Omega }$ with the corresponding parameter *v* = 0.15. Similarly, while the value of ${R}_{8}$ is set as ${R}_{8}=$ 13.33 $\mathrm{k\Omega }$ for the corresponding parameter $\nu$ =0.3, the phase portraits of chaotic attractors are demonstrated in Fig. 11c,f. Obviously, the simulation results of the circuit state Eq. (16) illustrated in Fig. 11 are similar to the theoretical numerical phase trajectories depicted in Fig. 7.

## Difference synchronization of chaotic systems

### Difference synchronization scheme

The difference synchronization scheme consists of two master systems and one slave system, where the master systems are defined as

11 $$\begin{array}{c}\dot{x}=Ax+F\left(x\right) ,\end{array}$$

12 $$\begin{array}{c}\dot{y}=By+G\left(y\right) ,\end{array}$$

and the slave system is considered as

13 $$\begin{array}{c}\dot{z}=Cz+H\left(z\right)+U\left(x,y,z\right) ,\end{array}$$

where $x={[{x}_{1}\left(t\right),{x}_{2}\left(t\right),{\dots ,x}_{n}(t)]}^{T}$, $y={[{y}_{1}\left(t\right),{y}_{2}\left(t\right),{\dots ,y}_{n}(t)]}^{T}$, $z={[{z}_{1}\left(t\right),{z}_{2}\left(t\right),{\dots ,z}_{n}(t)]}^{T}$ are state vectors of master systems and slave system, $F(x),G(y),H(z):$ R $\to R$ are the continuous vector functions and $U\left(x,y,z\right):R\times R\times R\to R$ is a controller which is going to be designed using feedback control technique.

#### Definition

The master systems and the slave system are said to be difference synchronization, if there exist three constant matrices ${M}_{1},{M}_{2},{M}_{3}\in R$ satisfying $\underset{t\to \infty }{\mathrm{lim}}\Vert {M}_{3}z-({M}_{2}y-{M}_{1}x)\Vert =0$ where ${M}_{3}\ne 0$ and $\Vert \cdot \Vert$ represent the norm of the matrix.

*Case 1* If constant matrices ${M}_{3}\ne 0$, ${M}_{2}\ne 0$, and ${M}_{1}=0$, the difference synchronization degenerates into complete synchronized mode.

*Case 2* If constant matrices ${M}_{3}\ne 0$, ${M}_{2}=0$, and ${M}_{1}\ne 0$, the difference synchronization degenerates into anti-synchronized mode.

### Difference synchronization of three nonidentical 3D chaotic systems

According to Lyapunov stability principle, the linearization method is used to determine the stability of the system (3). We convert Eq. (3) to

14 $$\begin{array}{c}\left(\begin{array}{c}\dot{x}\\ \dot{y}\\ \dot{z}\end{array}\right)=\left(\begin{array}{c}f\left(x\right)\\ f\left(y\right)\\ f\left(z\right)\end{array}\right)=AX+B,\end{array}$$

where $X={(x,y,z)}^{\tau }$. According to Taylor expansion theorem to obtain

15 $$\begin{array}{c}\left(\begin{array}{c}\dot{x}\\ \dot{y}\\ \dot{z}\end{array}\right)=\frac{\partial f}{\partial X}{|}_{X=0}\cdot X=\left(\begin{array}{ccc}0& 1& 0\\ -1& 0.5& 0\\ 0& 0& 0\end{array}\right)\left(\begin{array}{c}x\\ y\\ z\end{array}\right)+\left(\begin{array}{c}0\\ 0\\ -0.8\end{array}\right).\end{array}$$

The characteristic equation of coefficient matrix *A* can be derived as:

16 $$\begin{array}{c}{\lambda }^{3}{-0.5\lambda }^{2}+\lambda =0,\end{array}$$

where $\lambda$ is the eigenvalues of Eq. (16).

The eigenvalues of coefficient matrix *A* are ${\lambda }_{1}=0$, ${\lambda }_{\mathrm{2,3}}=(1\pm \sqrt{15}i)/4$. The system is unstable because of the positive real part of the eigenvalues ${\lambda }_{\mathrm{2,3}}$.

To formulate the difference synchronization method, systems (1) and (2) are considered as two master systems, and the slave system with control functions is specified by

17 $$\begin{array}{c}\left\{\begin{array}{l}\dot{{x}_{3}}={y}_{3}-2{x}_{3}{z}_{3}+{u}_{1}\left(t\right) ,\\ \dot{{y}_{3}}={-x}_{3}+0.5\left(1-{x}_{3}^{2}\right){y}_{3}-0.5{y}_{3}{z}_{3}+{u}_{2}(t)\\ \dot{{z}_{3}}={\nu x}_{3}^{2}+{0.1x}_{3}{y}_{3}-0.8+{u}_{3}\left(t\right) ,\end{array}\right.,\end{array}$$

where ${u}_{1}(t)$, ${u}_{2}(t)$ and ${u}_{3}(t)$ are the controllers need to be designed. Letting the matrices ${M}_{3}=diag({m}_{31},{m}_{32},{m}_{33})$, ${M}_{2}=diag\left({m}_{21},{m}_{22},{m}_{23}\right)$ and ${M}_{1}=diag({m}_{11},{m}_{12},{m}_{13})$, then error functions can be defined as follows:

18 $$\begin{array}{c}\left\{\begin{array}{l}{e}_{1}={m}_{31}{x}_{3}-{m}_{21}{x}_{2}+{m}_{11}{x}_{1},\\ {e}_{2}={m}_{32}{y}_{3}-{m}_{22}{y}_{2}+{m}_{12}{y}_{1},\\ {e}_{3}={m}_{33}{z}_{3}-{m}_{23}{z}_{2}+{m}_{13}{z}_{1}.\end{array}\right.\end{array}$$

By deriving the error functions (18), we can derive the error systems

19 $$\begin{array}{c}\left\{\begin{array}{l}\dot{{e}_{1}}={e}_{2}-2{m}_{31}{x}_{3}{z}_{3}+{m}_{31}{u}_{1}\left(t\right),\\ \dot{{e}_{3}}=-{e}_{1}+0.5{m}_{32}{y}_{3}-0.5{m}_{32}{x}_{3}^{2}{y}_{3}-0.5{m}_{32}{y}_{3}{z}_{3}+{m}_{22}{y}_{2}{z}_{2}-{m}_{12}{y}_{1}{z}_{1}+{m}_{32}{u}_{2}\left(t\right),\\ \dot{{e}_{3}}=0.1{m}_{33}{x}_{3}{y}_{3}+\nu {m}_{33}{x}_{3}^{2}-0.8{m}_{33}+b{m}_{23}-0.5{m}_{23}{x}_{2}^{2}-{m}_{23}{x}_{2}{y}_{2}+{m}_{13}{y}_{1}^{2}-a{m}_{13}+{m}_{33}{u}_{3}\left(t\right).\end{array}\right.\end{array}$$

The control functions are acquired while simplifying the linear term of the error system and adding linear feedback controllers:

20 $$\begin{array}{c}\left\{\begin{array}{l}{u}_{1}\left(t\right)=\frac{1}{{m}_{31}}\left(2{m}_{31}{x}_{3}{z}_{3}+{k}_{1}{e}_{1}\right),\\ {u}_{2}\left(t\right)=\frac{1}{{m}_{32}}\left(-0.5{m}_{32}{y}_{3}+0.5{m}_{32}{x}_{3}^{2}{y}_{3}+0.5{m}_{32}{y}_{3}{z}_{3}-{m}_{22}{y}_{2}{z}_{2}+{m}_{12}{y}_{1}{z}_{1}+{k}_{2}{e}_{2}\right),\\ {u}_{3}\left(t\right)=\frac{1}{{m}_{33}}\left(-0.1{m}_{33}{x}_{3}{y}_{3}-\nu {m}_{33}{x}_{3}^{2}+0.8{m}_{33}-b{m}_{23}+0.5{m}_{23}{x}_{2}^{2}+{m}_{23}{x}_{2}{y}_{2}-{m}_{13}{y}_{1}^{2}+a{m}_{13}+{k}_{3}{e}_{3}\right).\end{array}\right.\end{array}$$

Putting the control functions into error system, the error system is reduced to

21 $$\begin{array}{c}\left\{\begin{array}{l}\dot{{e}_{1}}={{k}_{1}{e}_{1}+e}_{2},\\ \dot{{e}_{3}}=-{e}_{1}+{k}_{2}{e}_{2},\\ \dot{{e}_{3}}={k}_{3}{e}_{3}.\end{array}\right.\end{array}$$

The Jacobian matrix of the linear error system (18) is

22 $$\begin{array}{c}J=\left[\begin{array}{ccc}{k}_{1}& 1& 0\\ -1& {k}_{2}& 0\\ 0& 0& {k}_{3}\end{array}\right].\end{array}$$

Following the criteria of Routh–Hurwitz, the error system is stabilized if the eigenvalues of the Jacobian matrix are negative, so three considered chaotic coupled systems would achieve differential synchronization. By calculation, the eigenvalues of Jacobian matrix (22) are ${\lambda }_{1}={k}_{3}$, ${\lambda }_{2}=({k}_{1}+{k}_{2}+\sqrt{{\left({k}_{1}-{k}_{2}\right)}^{2}-4})/2$, ${\lambda }_{3}=({k}_{1}+{k}_{2}-\sqrt{{\left({k}_{1}-{k}_{2}\right)}^{2}-4})/2$, where ${k}_{1}$, ${k}_{2}$,${k}_{3}$ are feedback factors.

If feedback factors satisfy

23 $$\begin{array}{c}\left\{\begin{array}{l}{k}_{1}+{k}_{2}<0,\\ \left|{k}_{1}-{k}_{2}\right|<2\\ {k}_{3}<0,\end{array}\right.,\end{array}$$

the difference synchronization among chaotic systems (1), (2), and (17) will be realized.

### Numerical simulation results

**Figure 12:**
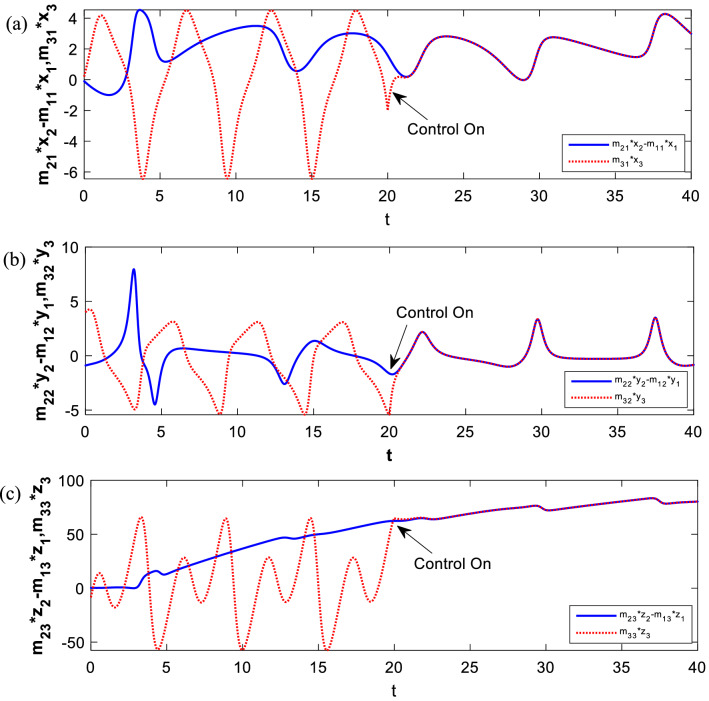
State trajectories of difference synchronization between (**a**) ${m}_{21}{x}_{2}-{m}_{11}{x}_{1}$ and ${m}_{31}{x}_{3}$, (**b**) ${m}_{22}{y}_{2}-{m}_{12}{y}_{1}$ and ${m}_{32}{y}_{3}$, (**c**) ${m}_{23}{z}_{2}-{m}_{13}{z}_{1}$ and ${m}_{33}{z}_{3}$.

To verify effectiveness of the difference synchronization, the fourth-order Runge–Kutta method is used to solve the equations in numerical simulation. Considering the parameters of the master Sprott A system and the slave NE8 system are taken as *a* = 1 and *b* = 1.47, the initial conditions are set as (0, 0.1, 0) and (− 0.1, − 1, 0.3), respectively. For initial condition (0.1, 2, 0.1), the parameter of the proposed system is considered as $\nu =0.3$. Thus, both the master systems and the slave system are chaotic in this situation according to the above analysis. In the absence of the controller defined by Eq. (20), the state trajectory of the master–slave system presents a dramatically chaotic state. Applying the controller at *t* = 20, selecting the feedback coefficient as ${k}_{1}={k}_{2}=- \, 4, {k}_{3}=- \, 1$, the master systems and salve system are difference synchronized in a short time using feedback control technology. Figure 12a–c demonstrate the state trajectory of the master–slave system before and after control.

**Figure 13:**
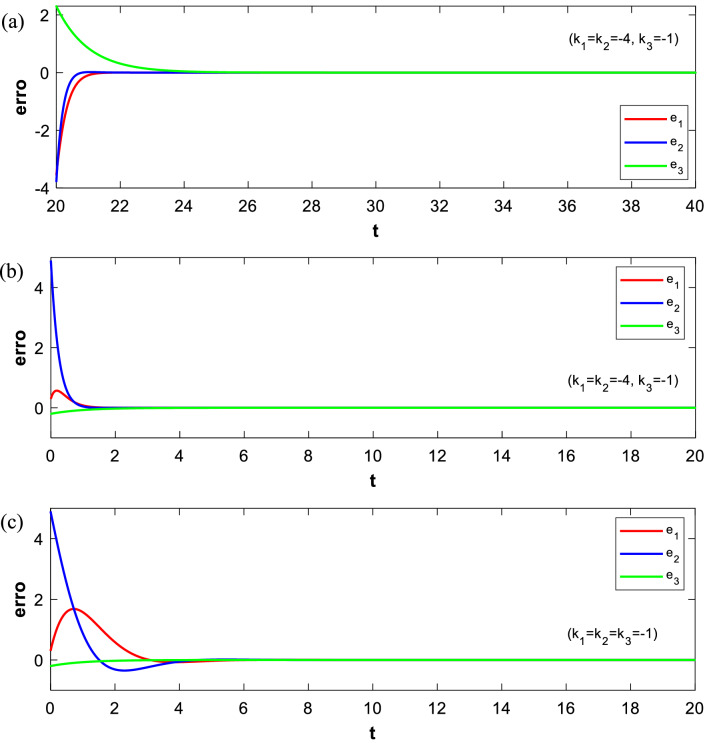
The trajectories of error functions with the controller activated at (**a**) *t* = 20 and ${k}_{1}={k}_{2}=-4, {k}_{3}=-1$. (**b**) *t* = 0 and ${k}_{1}={k}_{2}=-4, {k}_{3}=-1$. (**c**) *t* = 0 and ${k}_{1}={k}_{2}={k}_{3}=-1$.

The error curve in Fig. 13a converges to zero in a short time, predicting that the coupled system is differential synchronized. As shown in Fig. 13b, when turning on the control at *t* = 0, the synchronization time of systems will be significantly shortened, indicating that the synchronization time is affected by initial conditions. The feedback control coefficient of the error functions shown in Fig. 13c is adjusted to ${k}_{1}={k}_{2}={k}_{3}=-1$. It is obvious that the synchronization time of systems is significantly increased so that it can adapt to more practical engineering scenarios.

## Conclusion

In this paper, a novel three-dimensional symmetric chaotic system with multiple equilibrium points has been developed. The developed system is a kind of chaotic system with coexisting attractors. The dynamic behaviors including strange attractors, symmetric features, bifurcation diagram, maximal Lyapunov exponents, and local basins of attraction and bi-stability behaviors have been discussed. And we have given a clear route to investigate chaos behaviors by numeric analysis and obtain details behaviors of the chaotic system. To further confirm feasibility of the theoretical system, an electronic circuit emulating this chaotic system has been implemented by utilizing electronic simulation platform Multisim. All the results shown by the electronic circuit are closely consistent with those of numerical simulation. In addition, the feedback control method is used to achieve the difference synchronization between two mater systems Sprott A and the NE8 system with different structures. It indicates that the system proposed in this work can be practical for chaos-based engineering applications such as the design of self-excited oscillators and secure communication in future research.

## Data Availability

The data that support the findings of this study are available from the corresponding author on reasonable request.
